# Effects of Plyometric Training on Physical Performance: An Umbrella Review

**DOI:** 10.1186/s40798-022-00550-8

**Published:** 2023-01-10

**Authors:** Rafael L. Kons, Lucas B. R. Orssatto, Jonathan Ache-Dias, Kevin De Pauw, Romain Meeusen, Gabriel S. Trajano, Juliano Dal Pupo, Daniele Detanico

**Affiliations:** 1 Department of Physical Education, Faculty of Education, Federal University of Bahia, Salvador, Bahia 40110-100 Brazil; 2 School of Exercise and Nutrition Sciences, Faculty of Health, Queensland University of Technology (QUT), Brisbane, QLD Australia; 3 Research Group on Technology, Sport and Rehabilitation, Catarinense Federal Institute - IFC, Araquari, Brazil; 4 Human Physiology and Sports Physiotherapy Research Group and Brussels Human Robotics Research Center (BruBotics), Vrije Universiteit Brussel, Pleinlaan 2, 1050 Brussels, Belgium; 5 Biomechanics Laboratory, Centre of Sports - CDS, Federal University of Santa Catarina, Florianópolis, Santa Catarina Brazil

**Keywords:** Vertical jump, Motor actions, Sports performance, Muscle power

## Abstract

**Background:**

Plyometric training can be performed through many types of exercises involving the stretch-shortening cycle in lower limbs. In the last decades, a high number of studies have investigated the effects of plyometric training on several outcomes in different populations.

**Objectives:**

To systematically review, summarize the findings, and access the quality of published meta-analyses investigating the effects of plyometric training on physical performance.

**Design:**

Systematic umbrella review of meta-analyses.

**Data Sources:**

Meta-analyses were identified using a systematic literature search in the databases PubMed/MEDLINE, Scopus, SPORTDiscus, Web of Science, Cochrane Library and Scielo.

**Eligibility Criteria for Selecting Meta-analyses:**

Meta-analyses that examined the effects of plyometric training on physical fitness in different populations, age groups, and sex.

**Results:**

Twenty-nine meta-analyses with moderate-to-high methodological quality were included in this umbrella review. We identified a relevant weakness in the current literature, in which five meta-analyses included control group comparisons, while 24 included pre-to-post-effect sizes. Trivial-to-large effects were found considering the effects of plyometric training on physical performance for healthy individuals, medium-trivial effects for the sports athletes’ groups and medium effects for different sports athletes’ groups, age groups, and physical performance.

**Conclusion:**

The available evidence indicates that plyometric training improves most related physical fitness parameters and sports performance. However, it is important to outline that most meta-analyses included papers lacking a control condition. As such, the results should be interpreted with caution.

*PROSPERO number*: CRD42020217918.

**Supplementary Information:**

The online version contains supplementary material available at 10.1186/s40798-022-00550-8.

## Key Points

1. The available meta-analyses suggest that plyometric training induces trivial-to-large effects on physical performance for healthy people, and enhanced performance for athletes from different sports (e.g., vertical jump height, sprint time and muscle strength).
2. This umbrella review reveals that most meta-analyses include within-subject designs without control group comparisons.
3. Future original studies should include control groups in their experimental design to support the effects of plyometric training on physical and sports performance.

## Introduction

Plyometric training is broadly used to improve physical performance in many sports activities involving sprinting, jumping and change of direction ability [1–6]. It usually involves exercises that use the stretch-shortening cycle (SSC), in which a lengthening movement (eccentric) is quickly followed by a shortening movement (concentric) [7, 8]. The effective use of the SSC is related to the contributions of different mechanisms, such as the accumulation of elastic energy [7], pre-load [9], increase of the time to muscle activation [10], muscle history dependence (force enhancement) [11], stretch-reflexes [12] and muscle–tendon interactions [13] that facilitate greater mechanical work production in subsequent concentric muscle actions [14, 15].

The term “plyometric” first appeared in the work of the Russian researcher Zaciorski in 1966 [16]. Zaciorski proposed the term plyometric, considering that in these types of exercises involving SSC, the tension expressed by a group of muscle measured externally (“metron”) is higher (“plio”) than the muscle tension expressed when using other procedures, e.g., isometric exercise [16]. Different types of classifications for plyometric exercises have been used in the last seven decades. The first form of classification was proposed by Verkhoshanski [17], in which plyometric exercises were classified as impact (with some additional external load) and non-impact (without additional external load). More recently, plyometric training has been classified as traditional (e.g., jumps in place, standing jumps, multiple hops and jumps, bounds and drop jumps), assisted (when the exercise is assisted by an elastic band, for example) and resisted (when the exercises are performed under varied external conditions like water, sand and additional external loads) [18].

Over the last decades, numerous experimental studies have been suggesting positive effects of plyometric training on physical capacities such as muscle strength, muscle power, explosive strength and even endurance performance [19, 20]) and on performance of sport tasks such as sprint time, change of direction ability and jump performance [19, 21–23]. Changes in the neural and muscle mechanical properties (e.g., musculotendinous stiffness and architecture) [19, 20, 24] are also reported with plyometric training and may explain the improvements in the aforementioned physical capacities. The significant number of publications investigating the effects of plyometric training on physical capacities has grown widely, as have systematic reviews with meta-analysis studies. Especially in the last 14 years, papers included a wide range of sports activities, ages, and physical performance outcomes. To summarize the current knowledge on the topic and to identify possible methodological limitations in published meta-analyses, an umbrella review might be conducted [25], as this kind of review is considered on the highest level of the evidence pyramid [26]. Umbrella reviews highlight findings from already published meta-analyses, providing the state of the art about a given overarching topic with a high number of publications. Thus, they can help the reader to understand the current strengths and limitations of the entire body of literature on a specific topic from different perspectives and applications.

This study aimed: (i) to systematically review the available meta-analytical evidence that has examined the effects of plyometric training on physical fitness performance (e.g., sprint time, change of direction, maximal strength, muscle power and explosive strength, vertical or horizontal jump and specifying additional outcomes, such as endurance, high intermittent running performance, kicking performance, balance, and Yo-Yo intermittent recovery test) in different populations; (ii) to address the quality, strengths and limitations of the meta-analytical evidence considering plyometric training; and (iii) to identify current limitations in the literature and provide suggestions for future research. Our findings may be useful for coaches, scientists, athletes and physical training practitioners in understanding the meaningful and clinical effects of plyometric training for different populations (athletes and non-athletes, male and female) and different age ranges (young and older adults).

## Methods

Our umbrella review was conducted in accordance with recommendations of Aromataris and colleagues [25] and addressed all items recommended in the PRISMA (Preferred Reporting Items for Systematic Reviews and Meta-Analyses) statement [26]. It was registered in the PROSPERO database with the number: CRD42020217918.

### Literature Search

We conducted a systematic literature search in the databases PubMed/MEDLINE, Scopus, SPORTDiscus, Web of Science, Cochrane Library and Scielo during February and May 2022. A Boolean search syntax was used (Additional file 1: Appendix 1). The reference list of each included meta-analysis was screened for titles to identify additional meta-analyses to be included in the umbrella review.

### Selection Criteria

The studies were selected based on a priori defined inclusion/exclusion criteria (PICOS = population, intervention, comparison, outcome, study design), as shown in Table 1. Four independent reviewers (RLK, LBRO, JDP and DD) screened potentially relevant articles by analyzing titles, abstracts and full texts of the respective articles to elucidate their eligibility. When the four reviewers did not reach an agreement concerning inclusion of an article, JAD adjudicated.

**Table 1:** Selection criteria used in this umbrella review

Category	Inclusion criteria	Exclusion criteria
Population	Healthy people, with no restrictions on sex, age or sports modalities. It includes youth, adults and elderly (over 50 years) who are physically healthy and athletes from different sports modalities, such as teams, individual and combat sports	People with health problems (e.g., injuries and recent surgery)
Intervention	Programs based on the plyometric training approach considering the use of lower and upper body, unilateral or bilateral bounds, jumps, throws, and hops that commonly utilize a pre-stretch or countermovement potentiating of the stretch-shortening cycle	Exercise interventions not involving plyometric jump training or exercise interventions involving plyometric jump delivered in conjunction with other training interventions (e.g., resistance training)
Comparator	Control group or control situation	No active control group or control situation (e.g., stretching group or strength)
Outcome	Direct measure of physical fitness e.g., performance parameters based on sprint time, change of direction, maximal strength, muscle power, explosive strength, vertical or horizontal jump and additional outcomes such as endurance, high intermittent running performance, kicking performance, balance, and Yo-Yo intermittent recovery test before and after the training intervention	Lack of baseline or follow-up data
Study design	A Systematic Reviews and meta-analysis or only meta-analysis	No meta-analysis

### Data Extraction

The following data were extracted from the included meta-analyses: (1) first author and year of publication; (2) the number and type of primary studies included in the meta-analysis; (3) the study characteristics and the number of included participants; (4) the respective physical fitness outcome; (5) effect sizes and the equations used to compute effect sizes with their respective confidence intervals (CI). Data were extracted and cross-checked for accuracy by RLK, LBRO, JAD, JDP and DD.

### Evaluation of the Methodological Quality

Meta-analyses of randomized controlled trials and controlled studies are subject to different sources of bias. Therefore, it is important that readers have the option to distinguish between low- and high-quality meta-analyses. The methodological quality of the included meta-analyses was independently assessed by three reviewers (RLK, LBRO and JAD) using the validated AMSTAR 2 (A Measurement Tool to Assess Systematic Reviews) checklist [27]. This checklist contains 16 items that include the literature search procedure, data extraction, quality assessment and statistical analyses of the meta-analyses (for more details, see Shea et al. [27]). Each item on this checklist was answered with a ‘yes’ (1 point), ‘partial yes’ (0.5 points) or ‘no’ (0 points). Based on the summary point scores (i.e., maximum 16 points), the meta-analyses were categorized as high quality if ≥ 80% of the possible score was achieved, moderate quality if 40–79% of the possible score was reached, or low quality if < 40% of the possible score was achieved [28].

### Data Interpretation

The use of one effect size measure makes this comparison straightforward. However, it is important to acknowledge that even if most of the included meta-analyses used the standardized mean difference (SMD) as an effect size measure, differences were found in the respective equations that were used to compute SMDs. For instance, some meta-analyses weighted single studies and/or conducted sample size adjustment (e.g., Hedges’ g). Therefore, we extracted the effect sizes for each included meta-analysis (Table 2). According to Cohen [29], the SMD values were classified as: < 0.20 as trivial, 0.20 ≤ SMD < 0.50 as small, 0.50 ≤ SMD < 0.80 as moderate, and SMD ≥ 0.80 as large effects.

**Table 2:** Included meta-analyses that examined the effects of plyometric training on physical fitness in different population groups

Study	Population/sport	*N* participants/	*N* Studies included	Statistical model	Physical fitness outcome	Effect size (95% CI, *p* value); (*p* value)	*I*^2^
Alfaro-Jimenez et al. [38]	Team sports —young and adults (e.g., basketball, handball, volleyball, football and netball)	*N* = 50	*N* = 31	Within-subject SMD (Hedges’ g)	Explosive strength	0.98 (0.77–1.19, *p* < 0.05); (*p* = n.a)	72%
Asadi et al. [35]	Youth athletes—practitioners and non-practitioners of sports	*N* = 46	*N* = 24	Within-subject SMD (Hedges’ g)	Change of direction	0.59 (-0.08—1.24, n.a); n.a	n.a
Asadi et al. [36]	Youth athletes—practitioners and non-practitioners of sports	*N* = 667	*N* = 16	Within-subject SMD (Hedges’ g)	Change of direction	0.96 (n.a, n.a); n.a	n.a
Behm et al. [37]	Healthy trained or untrained boys and girls	*N* = 1351	*N* = 107	Within-subject SMD (Hedges’ g)	Vertical jump height, sprint performance and lower body strength	*Jump measures* Total 0.69 (0.53–0.84, * p* < 0.001); (*p* < 0.001)	51%
						Trained Boys 0.67 (0.52–0.82, *p* < 0.001); (*p* < 0.05)	39%
						Untrained 0.80 (0.24–1.35, * p* < 0.001); (*p* = 0.005)	80%
						Children 0.74 (0.53–0.94, * p* < 0.001); (*p* < 0.001)	62%
						Adolescents 0.57 (0.37–0.77, * p* < 0.01); (*p* > 0.05)	14%
						*Sprint performance* Total 0.38 (0.23–0.53, p (*p* < 0.001); (*p* > 0.05)	12%
						Trained boys 0.32 (0.18–0.46, * p* < 0.001); (*p* > 0.05)	0%
						Untrained 1.19 (− 0.32 to 2.69, * p* < 0.001); (*p* < 0.001)	87%
						Children 0.47 (0.28–0.67, * p* < 0.001); (*p* > 0.05)	31%
						Adolescents 0.13 (− 0.17 to 0.44, * p* > 0.05); (*p* > 0.05)	0%
						*Lower body strength* Adolescents 0.16 (− 0.26 to 0.58, *p* = 0.59); (*p* > 0.05)	0%
Berton et al. [44]	Healthy individuals—trained or untrained men	*N* = 158	*N* = 7	Within-subject SMD	Vertical jump height	0.15 (− 0.30 to 0.60, *p* = 0.51); 21% (*p* = 0.97)	21%
de Villarreal et al. [40]	Healthy individuals—with elite, high, medium and lower levels of fitness	*N* = 122	*N* = 56	Within-subject SMD (Hedges’ g)	Vertical jump height	Squat jump 0.79 (n.a, n.a); n.a	n.a
						CMJ 0.74 (n.a, n.a); n.a	n.a
						Drop jump 0.71 (n.a, n.a); n.a	n.a
						Sargent jump 0.57 (n.a, n.a); n.a	n.a
de Villarreal et al. [41]	Healthy individuals—with elite, high, medium and lower levels of fitness	*N* = 24	*N* = 15	Within-subject SMD (Hedges’ g)	Strength performance	0.97 (n.a, n.a); n.a	n.a
de Villarreal et al. [42]	Healthy individuals—with elite, high, medium and lower levels of fitness	*N* = 41	*N* = 26	Within-subject SMD (Hedges’ g)	Sprint time	0.37 (n.a, n.a); n.a	n.a
Kayantas et al. [46]	Athletes in general sports (e.g., basketball and football)	*N* = 1201	*N* = 6	Within-subject SMD	Speed parameters	0.67 (0.38–0.96, * p* < 0.001); (*p* < 0.007)	68%
Kayantas et al. [39]	Athletes in general sports (e.g., judo, basketball, volleyball, handball, football and wrestling)	*N* = 362	*N* = 11	Within-subject SMD	Muscular strength	0.40 (0.19–0.61, * p* < 0.001); (*p* = 0.36)	7%
Makaruk et al. [18]	Healthy individuals—age > 18 years	*N* = 602	*N* = 11	Within-subject SMD (Hedges’ g)	Vertical jump height	Traditional Plyometric 0.68 (0.37–0.99, * p* < 0.001); (*p* = 0.16)	31%
						Assisted Plyometric 0.70 (0.20–1.20, *p* = 0.006); (*p* = 0.94)	0%
						Resisted Plyometric 0.48 (0.17–1.19, *p* = 0.002); (*p* = 0.14)	33%
Markovic et al. [30]	Healthy individuals—athletes and non-athletes	*N* = 1024	*N* = 43	Experimental vs. Control SMD	Vertical jump height	Squat jump 0.44 (0.15–0.72, n.a); (n.a)	33%
						CMJ 0.88 (0.64–1.11, n.a); (n.a)	11%
						CMJ with the arm swing 0.71 (0.49–0.93, n.a); (n.a)	26%
						Drop jump 0.62 (0.18–1.05, n.a); (n.a)	20%
Moran et al. [31]	Older healthy individuals’ adults (> 50)	*N* = 444	*N* = 9	Experimental versus control SMD	Lower limbs power	0.66 (0.33–0.98, *p* = 0.02); (*p* < 0.001)	51%
Moran et al. [32]	Healthy trained or untrained girls	(8–18 years); *N* = 452	*N* = 14	Experimental versus control SMD (Hedges’ g)	Vertical jump height	0.57 (0.21–0.93; * p* < 0.01); (*p* < 0.001)	68%
Moran et al. [45]	Healthy individuals—Untrained and trained	*N* = n.r	*N* = 9	Within-subject SMD	Vertical and horizontal jump performance	Horizontal plyometric training Horizontal jump 1.05 (0.38–1.72, n.a); (*p* = 0.002)	73%
						Vertical Jump 0.74 (0.08–1.40, n.a); (*p* = 0.03)	75%
						Vertical plyometric training Horizontal jump 0.84 (0.37–1.31, n.a); (*p* = 0.0005)	52%
						Vertical jump 0.72 (0.02–1.43, n.a); (*p* = 0.04)	78%
Ozdemir et al. [47]	Athletes in general sports (e.g., badminton, basketball, football, wrestling, handball and volleyball)	*N* = 40	*N* = 43	Within-subject SMD	Vertical jump performance	0.68 (0.57–0.80, *p* < 0.001); (*p* < 0.001)	49%
Ramirez-Campillo et al. [54]	Handball players	*N* = 129	*N* = 5	Within-subject SMD	Vertical jump height	2.15 (0.95–3.36, *p* < 0.001); (*p* < 0.001))	51%
Ramirez-Campillo et al. [51]	Volleyball players	*N* = 346	*N* = 14	Within-subject SMD	Vertical jump height	2.07 (1.22–2.93, *p* < 0.001); (*p* = 0.087)	34%
Ramirez-Campillo et al. [50]	Team sports (e.g., soccer, volleyball, basketball and futsal)	*N* = 278	*N* = 14	Within-subject SMD	Vertical jump height	0.73 (0.45–1.02, *p* < 0.001); (*p* = 0.22)	18%
Ramirez-Campillo et al. [55]	Female soccer players	*N* = 99	*N* = 8	Within-subject SMD	Vertical jump height	1.01 (0.36–1.66, *p* = 0.002); (*p* = 0.33)	13%
Ramirez-Campillo et al. [53]	Basketball players	*N* = 818	*N* = 32	Within-subject SMD	Vertical jump power, countermovement jump with arm swing height, Countermovement jump height, squat jump height, drop jump height, horizontal jump distance, < 10-m linear sprint time, > 10-m linear sprint time, < 40-m change-of-direction performance time, > 40-m change-of-direction performance time, dynamic balance, static balance, maximal strength, hamstring/quadriceps strength ratio at 60°/s, hamstring/quadriceps strength ratio at ≥ 120°/s	*Jumping* Vertical jump power, 0.45 (0.07–0.84, *p* = 0.021); (*p* = 0.32)	0%
						Countermovement jump with arm swing height 1.24 (0.72–1.75, < 0.001); (*p* < 0.001)	71%
						Countermovement jump height 0.88 (0.55–1.22, *p* < 0.001); (*p* = 0.071)	67%
						Squat jump height 0.80 (0.47–1.14, *p* < 0.001); (*p* = 0.008)	52%
						Drop jump height 0.53 (0.25–0.80, *p* < 0.001); (*p* = 0.567)	0%
						Horizontal jump distance 0.65 (− 0.02 to 1.31, *p* < 0.001); (p = 0.008)	80%
						***Sprint*** < 10-m linear sprint time 1.67 (0.32–3.03, *p* = 0.016); (*p* = 0.307)	85%
						> 10-m linear sprint time 0.92 (0.40–1.44, *p* < 0.001); (*p* = 0.061)	74%
						< 40-m change-of-direction performance time 1.15 (0.75–1.55, *p* < 0.001); (*p* = 0.189)	59%
						> 40-m change-of-direction performance time 1.02 (0.29–1.76, *p* = 0.006); (*p* = 0.272)	64%
						*Balance* Dynamic balance 1.16 (0.43–1.89, *p* = 0.002); (*p* = 0.586)	76%
						Static balance 1.48 (− 0.19–3.15, *p* = 0.002); (*p* = 0.252)	93%
						*Strength variables* Maximal strength 0.57 (0.07–1.07, *p* = 0.025); (*p* = 0.117)	38%
						Hamstring/quadriceps strength ratio at 60°/s − 0.10 (− 0.56 to − 0.36, *p* = 0.661); (*p* = 0.060)	23%
						Hamstring/quadriceps strength ratio at ≥ 120°/s − 0.04 (− 0.56 to 0.48, *p* = 0.885); (*p* = 0.785)	39%
Ramirez-Campillo et al. [52]	Volleyball players	*N* = 746	*N* = 18	Within-subject SMD (Hedges’ g)	Linear sprint speed, squat jump height, countermovement jump height, CMJ with arm swing, drop jump and spike jump height	Linear sprint speed 0.70 (0.31–1.09, *p* < 0.001); *p* = 0.609	46%
						Squat jump 0.56 (0.24–0.88, *p* = 0.001); *p* = 0.409	0%
						Countermovement jump 0.80 (0.37–1.22, *p* < 0.001); *p* = 0.270	66%
						Countermovement jump with arm swing, 0.63 (0.21–1.04, *p* = 0.003); *p* = 0.002	0%
						Drop jump 0.81 (0.15–1.47, *p* = 0.016); *p* = 0.496	37%
						Spike jump height 0.84 (0.36–1.32, *p* = 0.001); (*p* < 0.05)	0%
Sánchez et al. [56]	Female soccer players	*N* = 250	*N* = 10	Within-subject SMD (Hedges’ g)	Countermovement jump, drop jump, kicking performance, linear sprint, change of direction speed, and endurance	Countermovement jump 0.71 (0.20–1.23, *p* = 0.007); (*p* = 0.224)	62%
						Countermovement jump with Arm Swing 0.41 (-0.34–1.15, *p* = 0.28); (*p* = 0.452)	65%
						Drop jump 0.79 (0.12–1.47, *p* = 0.021); (*p* = 0.063)	73%
						Kicking performance 2.24 (0.13–4.36, *p* < 0.037); (*p* = 0.040)	89%
						Linear sprint 0.79 (0.39–1.18, *p* < 0.001); (*p* = 0.257)	38%
						Change of direction speed 0.73 (0.39–1.06, *p* < 0.001); (*p* = 0.813)	0%
						Endurance 0.60 (0.09–1.10, *p* = 0.020); (*p* = 0.328)	53%
Singla et al. [57]	Healthy individuals—practitioners and non-practitioners of sports	*N* = 287	*N* = 11	Within-subject SMD	Ball throwing velocity and distance. Upper body power and strength	Velocity 0.68 (0.01–1.36, *p* < n.a); (*p* = 0.07)	7%
						Distance 0.42 (− 0.07 to 0.92, *p* < n.a); (*p* = 0.17)	3%
						Power -0.08 (-0.45–0.29, *p* < n.a); (*p* = 0.45)	1%
						Strength 0.15 (-0.52–0.82, *p* < n.a); (*p* = 0.14)	4%
Slimani et al. [33]	Soccer players	*N* = 355	*N* = 10	Experimental vs. Control SMD	Vertical jump height	0.85 (0.47–1.23, *p* < 0.001); (*p* < 0.001)	68%
Sole et al. [48]	Individual sport athletes (e.g., runners, gymnastics, golfers, tennis, swimmers, throwers, fencers, cyclists and recreational resistance training)	*N* = 667	*N* = 26	Within-subject SMD (Hedges’ g)	Vertical jump, linear sprint, maximal strength, endurance performance	Vertical jump 0.49 (0.32–0.65, *p* < 0.001); (*p* < 0.117)	0%
						Linear sprint 0.23 (0.02–0.44, *p* = 0.032); (*p* = 0.518)	10%
						Maximal strength 0.50 (0.23–0.77, *p* < 0.001); (*p* = 0.004)	0%
						Sprint with change of direction 0.34 (− 0.19 to 0.87, *p* = 0.205); (*p* = 0.657)	70%
						Endurance performance 0.30 (0.03–0.57, *p* = 0.028); (*p* = 0.119)	11%
Stojanovic et al. [50]	Female general athletes (e.g., basketball, amateur soccer, elite runners, collegiate soccer players, hockey and volleyball players)	*N* = 437	*N* = 16	Within-subject SMD (Hedges’ g)	Countermovement jump without arm swing, countermovement jump with arm swing, squat jump, drop jump	Countermovement jump without arm swing 1.87 (0.73–3.01, n.a); (n.a)	75%
						Countermovement Jump with Arm Swing 1.31 (− 0.04 to 2.65, n.a); (n.a)	92%
						Squat jump 0.44 (− 0.09 to 0.97, n.a); (n.a)	0%
						Drop jump 3.62 (3.03–4.21, n.a); (n.a)	96%
Taylor et al. [43]	Healthy individuals trained sports practitioners	*N* = 188	*N* = 31	Within-subject SMD (Hedges’ g)	Vertical jump, Sprint (10, 20, 30 m) ability and high-intensity intermittent running performance	Vertical jump 0.33 (0.03—0.63), n.a); (n.a)	33%
						Sprint 10 m 0.42 (0.18–0.66, n.a); (n.a)	0%
						Sprint 20 m 0.49 (0.03–0.95 0.46, n.a); (n.a)	61%
						Sprint 30 m 1.01 (0.08–1.94 ± 0.93, n.a); (n.a)	47%
						Repeated sprint ability 0.62 (0.37–0.87, n.a); (n.a)	0%
						High intermittent running performance 0.61 (0.07–1.15; 0.54, n.a); (n.a)	56%
van de Hoef et al. [34]	Male soccer players	*N* = 564	*N* = 17	Experimental versus control SMD	Vertical jump, Sprint (5, 10, 15, 20, 30 m) CMJ vertical jump height performance, strength, agility and Yo-Yo Intermittent Recovery Test 1 and 2	Vertical jump (cm) 1.07 (0.13–2.00, n.a); (*p* = 0.46)	0%
						Sprint 5 m (s) 0.00 (− 0.02 to 0.02, n.a); (*p* = 0.98)	
						Sprint 10 m (s) 0.01 (− 0.01 to 0.04, n.a); (*p* = 0.23)	0%
						Sprint 15 m (s) 0.04 (− 0.03 to 0.12, n.a); (*p* = 0.17) Sprint 20 m (s)	27%
						0.05 (− 0.01 to 0.10, n.a); (*p* = 0.48)	46%
						Sprint 30 m (s) 0.05 (− 0.02 to 0.11, n.a); 0% (*p* = 0.53)	0%
						Strength (kg) 8.49 (− 10.64 to 27.61, n.a); (*p* < 0.001)	97%
						Agility (s) 0.01 (− 0.07 to 0.10, n.a); (*p* = 0.18)	34%
						Yo-Yo Intermittent Recovery Test 1 and 2 (cm) 120.74 (3.00–238.49, n.a); (*p* = 0.16)	42%

n.a = not assessed; n.r = not reported; SMD = standardized mean difference; *I*^2^ = percentage of total variability due to between-study heterogeneity

## Results

### Search Results

**Fig. 1:**
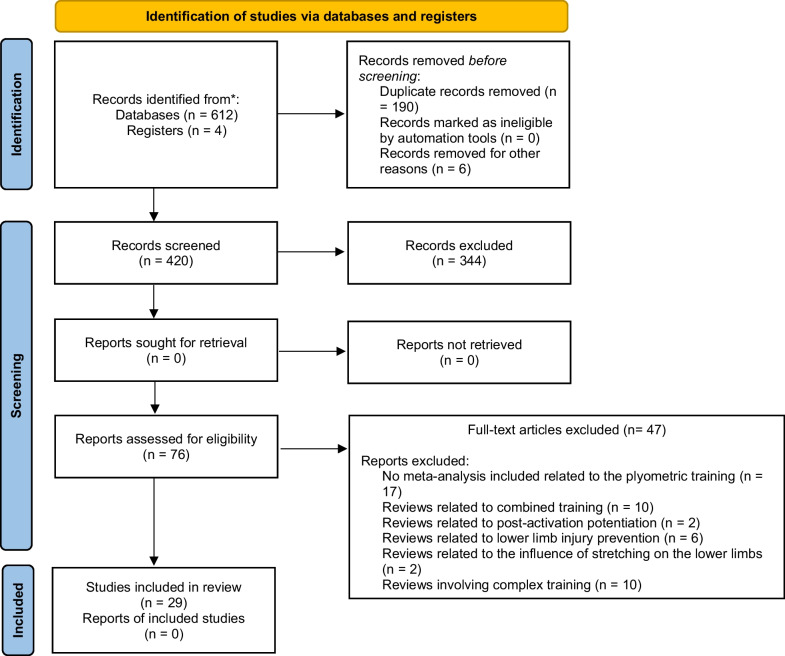
PRISMA flow diagram for the systematic search

The systematic search identified a total of 416 potentially relevant studies in the searched electronic databases after removing duplicates. Full text of 76 articles were read and 47 were excluded based on a priori defined selection criteria. Finally, 29 systematic reviews with meta-analyses were eligible for inclusion in this umbrella review. Figure 1 presents the PRISMA flow diagram for the systematic search. The publication dates of the meta-analyses included in this umbrella review ranged from 2009 to 2021.

### Characteristics of the Meta-analyses

The 29 included meta-analyses were published from 2007 to 2022 (Table 2). Five meta-analyses compared the effects of intervention to control group [30–34], while the other 24 compared within-intervention-group effects (i.e., pre- vs post-effect sizes). The number of included original studies ranged from 6 to 107 with an average of 22 original studies. Sample sizes included 24 to 2471 athletes of specific sports (e.g., volleyball, soccer, handball and basketball), groups of sports (e.g., team sports and individual sports), healthy people, and individuals from different age groups (i.e., young, young adults and older adults) (on average 459 participants). The chronological age of the included participants ranged from 15 to 71 years. Five meta-analyses included adolescents [35–39], ten meta-analyses involved healthy people [18, 30, 31, 40–45], three meta-analyses focused on athletes participating in general sports [39, 46, 47], one meta-analysis involved older adults (> 50 years) [31], one meta-analysis included female athletes participating in general sports [39] and one meta-analysis focused on individual sports athletes (e.g., runners, gymnasts, golfers, swimmers, tennis players, javelin, fencers and cyclists) [48]. When considering the sports modality, two meta-analyses included general team sports [38, 49] and one meta-analysis individual sports [48]. Within the team sports, two meta-analyses analyzed female soccer players [33, 50], two meta-analyses volleyball players [51, 52], two meta-analyses male soccer players, [33, 34] one meta-analysis basketball players [53], and one meta-analysis handball players [54] considering both sexes.

### Assessment of the Methodological Quality

The assessment of the methodological quality (AMSTAR 2) of the included meta-analyses is summarized in Table 3. The included articles received scores ranging from 12 to 84% of the maximum score (16 points). Twenty-two meta-analyses (75.9% of total articles included) [18, 30–33, 35, 37, 40–43, 45, 48, 49, 49–57] were rated as moderate quality, six were rated as low quality (20.7% of total articles included) [36, 38, 39, 43, 46, 47] and one was rated as high quality (3.4% of total articles included) [34]. The following criteria were not sufficiently addressed in the included meta-analyses: (*n* = 2) establish methods prior to conducting the meta-analysis (written protocol); (*n* = 3) explain the choice of study design for inclusion; (*n* = 7) provide a list of excluded studies to justify the exclusion; and (*n* = 10) report sources of funding for included studies.

**Table 3:** Results of the assessment of the methodological quality of the included meta-analyses using AMSTAR 2 (A measurement Tool to Assess systematic reviews)

Meta-analysis	AMSTAR 2 items																	
	1	2	3	4	5	6	7	8	9	10	11	12	13	14	15	16	Scores (%)	Quality
Alfaro-Jimenez et al. [38]	No	No	No	Partial yes	No	No	Yes	No	No	No	Yes	No	No	No	No	No	15	Low
Asadi et al. [35]	Yes	No	Yes	Partial yes	No	Yes	Partial Yes	Yes	Partial Yes	No	Yes	No	No	No	No	No	40	Moderate
Asadi et al. [36]	Yes	No	Yes	Partial yes	No	No	No	Yes	No	No	No	No	No	No	No	No	21	Low
Behm et al. [37]	Yes	No	Yes	Partial yes	No	Yes	Yes	Yes	No	No	Yes	No	No	No	No	No	40	Moderate
Berton et al. [44]	No	No	No	Partial yes	No	Yes	Partial Yes	Partial yes	No	No	No	No	No	Yes	No	No	21	Low
de Villarreal et al. [40]	Yes	No	Yes	Partial yes	Yes	Yes	Partial Yes	Yes	No	No	Yes	No	No	No	No	No	43	Moderate
de Villarreal et al. [41]	Yes	No	Yes	Partial yes	Yes	Yes	No	Yes	Partial yes	No	Yes	No	No	No	No	No	43	Moderate
de Villarreal et al. [42]	Yes	No	Yes	Partial yes	Yes	Yes	No	Yes	No	No	Yes	No	No	No	No	No	40	Moderate
Kayantas et al. [46]	No	No	No	No	No	No	No	No	Partial yes	No	Yes	No	Yes	Yes	Yes	No	28	Low
Kayantas et al. [39]	No	No	No	No	No	No	No	No	Partial yes	No	Yes	No	Yes	Yes	Yes	No	28	Low
Makaruk et al. [18]	Yes	No	No	Partial yes	Yes	Yes	Yes	Partial yes	Partial yes	No	Yes	No	No	Yes	Yes	No	53	Moderate
Markovic et al. [30]	Yes	No	Yes	Partial yes	No	No	No	Partial yes	Partial yes	No	Yes	No	Yes	Yes	Yes	No	46	Moderate
Moran et al. [31]	Yes	No	Yes	Partial yes	No	Yes	Yes	Partial yes	Partial yes	No	Yes	No	No	Yes	Yes	No	53	Moderate
Moran et al. [32]	Yes	No	No	Partial yes	Yes	Yes	Yes	Partial yes	No	No	Yes	Yes	Yes	Yes	No	No	56	Moderate
Moran et al. [45]	Yes	No	No	Partial yes	Yes	No	Yes	Partial yes	Yes	No	Yes	Yes	Yes	Yes	Yes	No	62	Moderate
Ozdemir et al. [47]	No	No	No	No	No	No	Yes	No	No	No	Yes	No	No	No	No	No	12	Low
Ramirez-Campillo et al. [54]	Yes	No	No	Partial yes	Yes	Yes	Partial yes	Yes	Yes	No	Yes	Yes	Yes	Yes	Yes	No	68	Moderate
Ramirez-Campillo et al. [51]	Yes	No	Yes	Partial yes	Yes	Yes	Partial yes	Yes	Yes	No	Yes	Yes	Yes	Yes	Yes	No	75	Moderate
Ramirez-Campillo et al. [50]	Yes	No	No	Partial yes	Yes	Yes	Partial yes	Yes	Yes	No	Yes	Yes	Yes	Yes	Yes	No	68	Moderate
Ramirez-Campillo et al. [55]	Yes	No	No	Partial yes	Yes	Yes	Partial yes	Yes	Yes	No	Yes	Yes	Yes	Yes	Yes	No	68	Moderate
Ramirez-Campillo et al. [53]	Yes	Yes	No	Partial yes	Yes	Yes	Yes	Yes	Yes	No	Yes	Yes	Yes	Yes	Yes	No	78	Moderate
Ramirez-Campillo et al. [52]	Yes	No	No	Partial yes	Yes	Yes	Yes	Yes	Partial yes	No	Yes	Yes	Yes	No	No	No	56	Moderate
Sánchez et al. [56]	Yes	No	Yes	Partial yes	No	No	Partial yes	Yes	Partial yes	No	Yes	Yes	Yes	Yes	No	No	53	Moderate
Singla et al. [57]	Yes	No	No	Partial yes	Yes	Yes	Partial yes	Yes	Partial yes	No	Yes	No	No	Yes	No	No	46	Moderate
Slimani et al. [33]	Yes	No	No	Partial yes	Yes	Yes	Partial yes	Partial yes	Partial yes	No	Yes	No	No	Yes	No	No	43	Moderate
Sole et al. [48]	Yes	No	No	Partial yes	No	No	Partial yes	Yes	Partial yes	No	Yes	Yes	Yes	Yes	Yes	No	53	Moderate
Stojanovic et al. [50]	Yes	No	No	Partial yes	Yes	Yes	Yes	Yes	Partial yes	No	Yes	Yes	No	No	No	No	50	Moderate
Taylor et al. [43]	Yes	No	No	Partial yes	Yes	Yes	Yes	Yes	Partial yes	No	Yes	No	No	Yes	No	No	50	Moderate
van de Hoef et al. [34]	Yes	Yes	Yes	Partial yes	Yes	Yes	Yes	Yes	Yes	No	Yes	Yes	Yes	Yes	Yes	No	84	High

1 = Word research question and inclusion criteria according to PICOS (population, intervention, comparison, outcome, study design), 2 = Establish methods prior to the conduct of the meta-analyses (written protocol), 3 = Explain the choice of study design for inclusion, 4 = Use comprehensive literature search strategy, 5 = Perform study selection in duplicate, 6 = Perform data extraction in duplicate, 7 = Provide a list of excluded studies to justify the exclusion, 8 = Describe the included studies in detail, 9 = Assess the risk of bias, 10 = Reported sources of funding for included studies, 11 = Use appropriate method for statistical combination of results, 12 = Assess the potential impact of risk of bias for included studies, 13 = Account for risk of bias while interpreting/discussing the results, 14 = Explain/discuss any heterogeneity, 15 = Assess publication bias and discuss its impact on the results, 16 = Report potential sources of conflict of interest and describe any funding

### Effect of Plyometric Training on Sprint Time

**Fig. 2:**
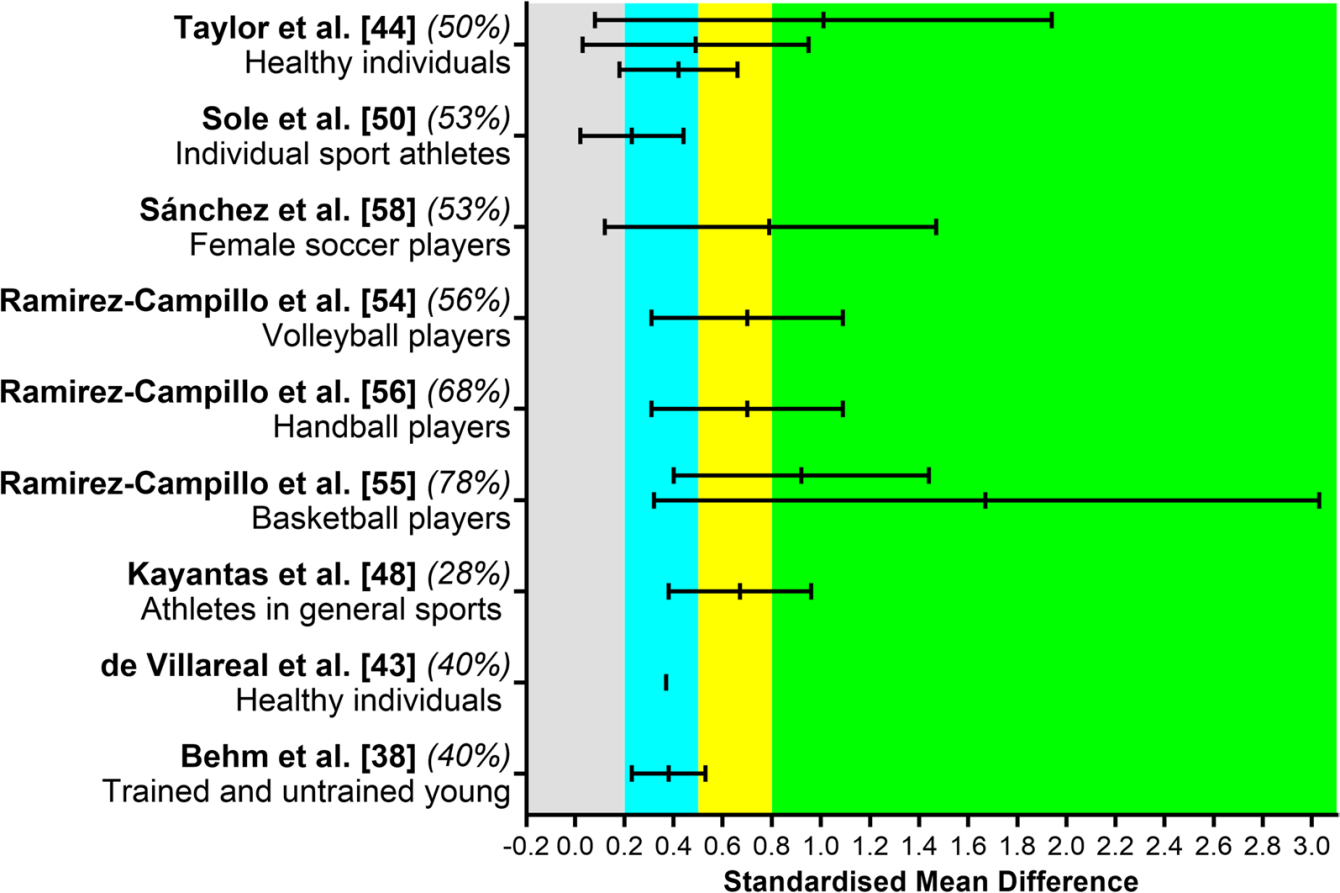
Summary of standardized mean difference and 95% confidence intervals reported in meta-analyses comparing the baseline to post-plyometric training changes in sprint time. Author name and year are followed by the quality of the studies score ranked by AMSTAR 2. Positive values represent improved performance effects. Each colored area represents a different magnitude of effect: gray = trivial, blue = small, yellow = moderate, and green = large effects. De Villareal et al. [42] 95% confidence interval is not clearly described in their manuscript; therefore, we reported standardized mean difference only. Taylor et al. [43] reported results from 30-, 20-, and 10-m sprints, presented in the respective order. Ramirez-Campillo et al. [53] reported results from > 10- and < 10-m sprints, presented in the respective order

Nine meta-analyses identified positive effects and one meta-analysis reported no effect of plyometric training on sprint time. Figure 2 summarizes the effects in terms of standardized mean difference between baseline and post-training values. In non-athlete individuals, there was a small effect for 10-m and 20-m sprint time, a large effect for 30-m sprint time [43], and a small effect for general sprint time [42] (Fig. 2). For young (< 18 years old) participants, there was a small effect when analyzing the total effect in trained and untrained participants [37]. When analyzing meta-analyses that included only athletes, there was a small effect observed for individual sport [48], but a moderate effect for athletes in general sports [46]. A moderate effect was observed for female soccer players [56], handball players [54], and volleyball players [51], while a large effect size was observed for basketball players (for sprints > or < than 10 m) [53]. There was an unclear effect on 5-, 10-, 15-, 20-, and 30-m sprint time in male soccer players [34].

### Effect of Plyometric Training on Change of Direction Ability

**Fig. 3:**
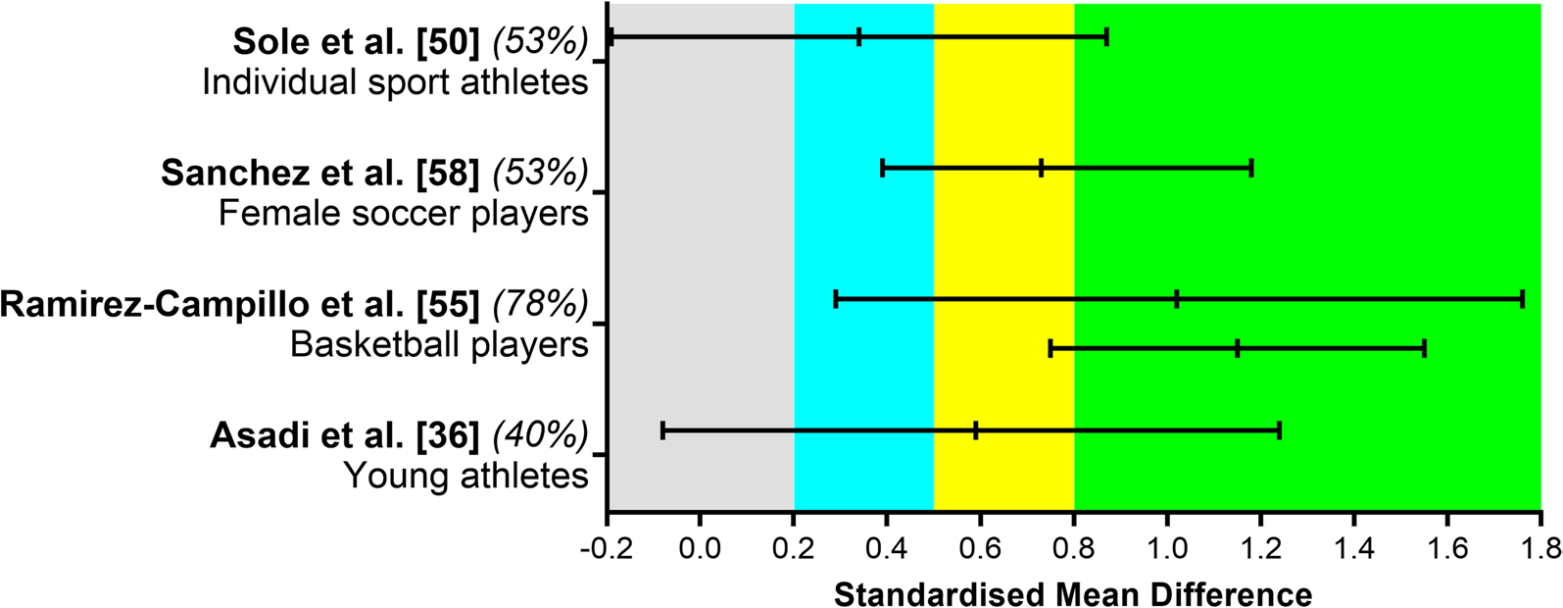
Summary of standardized mean difference and 95% confidence intervals reported in meta-analyses comparing the baseline to post-plyometric training changes on change of direction performance. Author name and year are followed by the quality of the studies score ranked by AMSTAR 2. Positive values represent improved performance effects and negative values detrimental effects. Each colored area represents a different magnitude of effect: gray, trivial; blue, small; yellow, moderate; and green, large effects. Ramirez-Campillo et al. [53] reported results from > 40- and < 40-m testing distances, presented in the respective order

Figure 3 summarizes the effects observed on change of direction in the four studies reporting standardized mean difference comparing baseline and post-training values. Two meta-analyses reported improvements and two found unclear differences on change of direction performance after plyometric training. A large effect was observed in basketball players (for running distances shorter or longer than 40 m) [53] and a moderate effect for female soccer players [56]. Unclear effect was observed for individual sport athletes [48] and young athletes [36]. Also, one study reported an unclear effect in soccer players [34].

### Effect of Plyometric Training on Maximal Strength

**Fig. 4:**
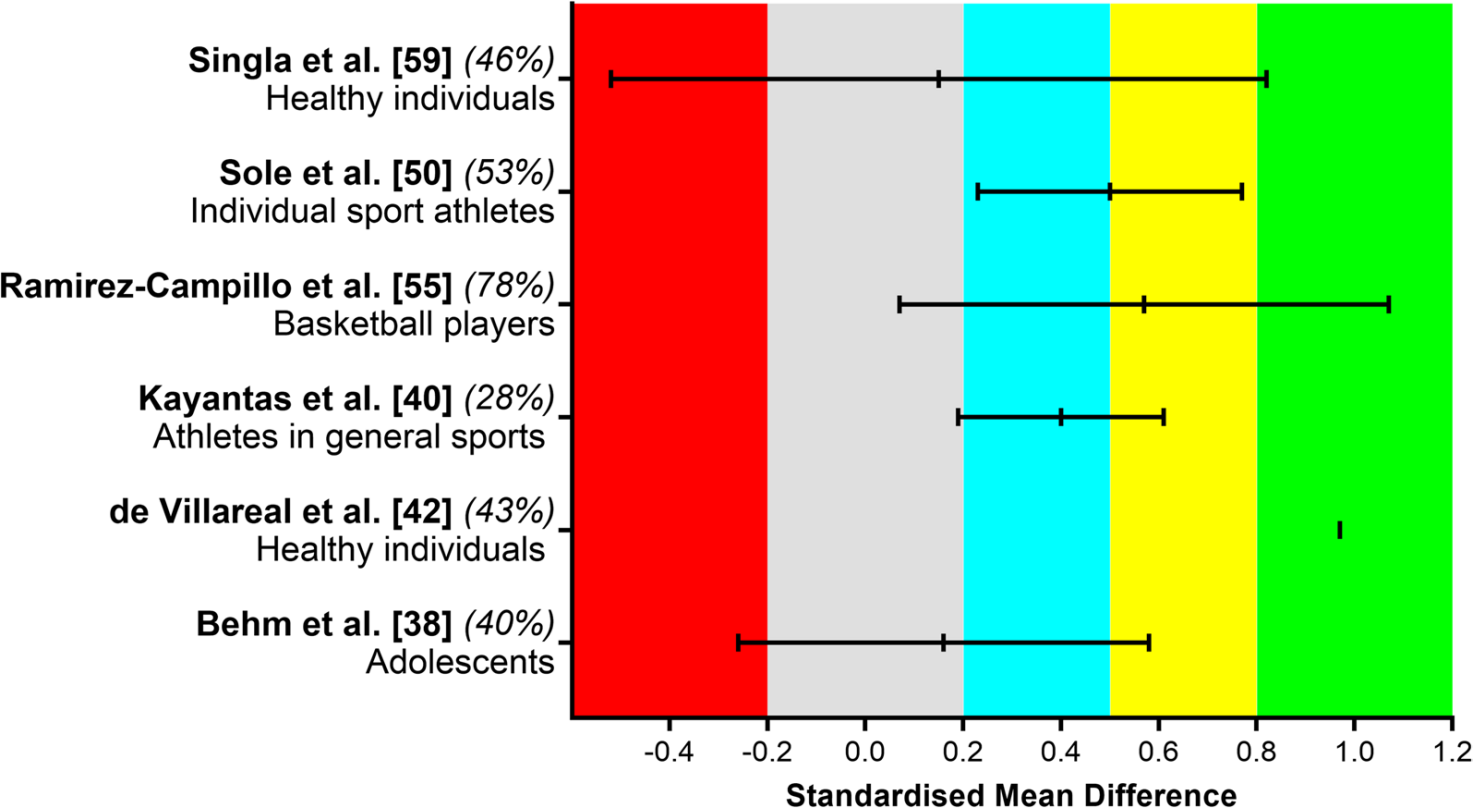
Summary of standardized mean difference and 95% confidence intervals reported in meta-analyses comparing the baseline to post-plyometric training changes on muscular strength performance. Author name and year are followed by the quality of the studies score ranked by AMSTAR 2. Positive values represent improved performance effects and negative values detrimental effects. Each colored area represents a different magnitude of effect: gray = trivial, blue = small, yellow = moderate, and green = large effects, while the red area represents detrimental effects. De Villareal et al. [42] did not clearly describe the 95% confidence interval; thus, we only reported standardized mean difference

Figure 4 summarizes the effects of plyometric training on muscular strength performance. Seven studies reported standardized mean difference comparing baseline and post-training values. Four meta-analyses reported positive effects and three reported unclear differences on muscular strength performance (1RM or isokinetic tests), for upper [57] or lower limb [48], after plyometric training. A large and unclear effect was observed for healthy individuals [41, 57] and also healthy adolescents [37], a moderate effect for basketball players [53] and individual sport athletes [48], and a small effect for athletes from general sports [39]. Also, one study reported an unclear effect in soccer players [34]. Only one study showed that an unclear effect was also observed for hamstring/quadriceps strength ratios at 60 and ≥ 120°/s in basketball players [53].

### Effect of Plyometric Training on Muscular Power and Explosive Strength

**Fig. 5:**
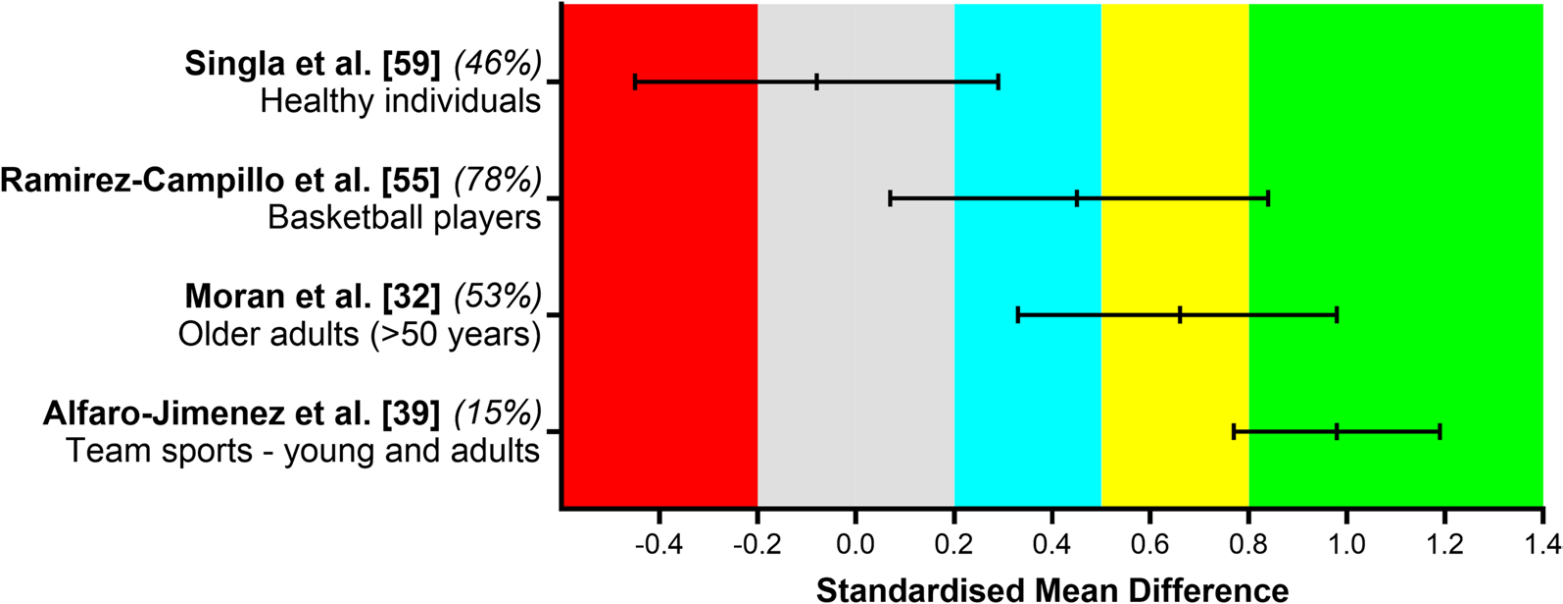
Summary of standardized mean difference and 95% confidence intervals reported in meta-analyses comparing the baseline to post-plyometric training changes on power or explosive muscular strength performance. Author name and year are followed by the quality of the studies score ranked by AMSTAR 2. Positive values represent improved performance effects and negative values detrimental effects. Each colored area represents a different magnitude of effect: gray = trivial, blue = small, yellow = moderate, and green = large effects, while the red area represents detrimental effects. Alfaro-Jimenez et al. [38] investigated the effects on explosive strength and the other authors on muscular power

There was a large effect observed for explosive strength in team sport athletes [38] For muscular power, there was a moderate effect for older adults [31], a small effect for basketball players [53], and an unclear effect for healthy individuals [57]. Figure 5 summarizes the effects observed on power and explosive muscular strength performance in the four studies reporting standardized mean difference comparing baseline and post-training values.

### Effect of Plyometric Training on Vertical and Horizontal Jump Performance

Several studies investigated the effects of plyometric training on squat jump, countermovement jump (with arm swing or hands on the hip), drop jump, Sargent jump, and/or spike jump performance (i.e., jump height). In summary, for healthy people an unclear-to-large effect was observed [30, 40, 43, 44]. Athletes from team sports, such as soccer [33, 34, 55, 56], volleyball [51, 52], basketball [53], handball [54], or when grouped as team sports [49], presented mostly moderate-to-large effects. Trained and untrained young individuals presented moderate effect sizes [34, 37].

**Fig. 6:**
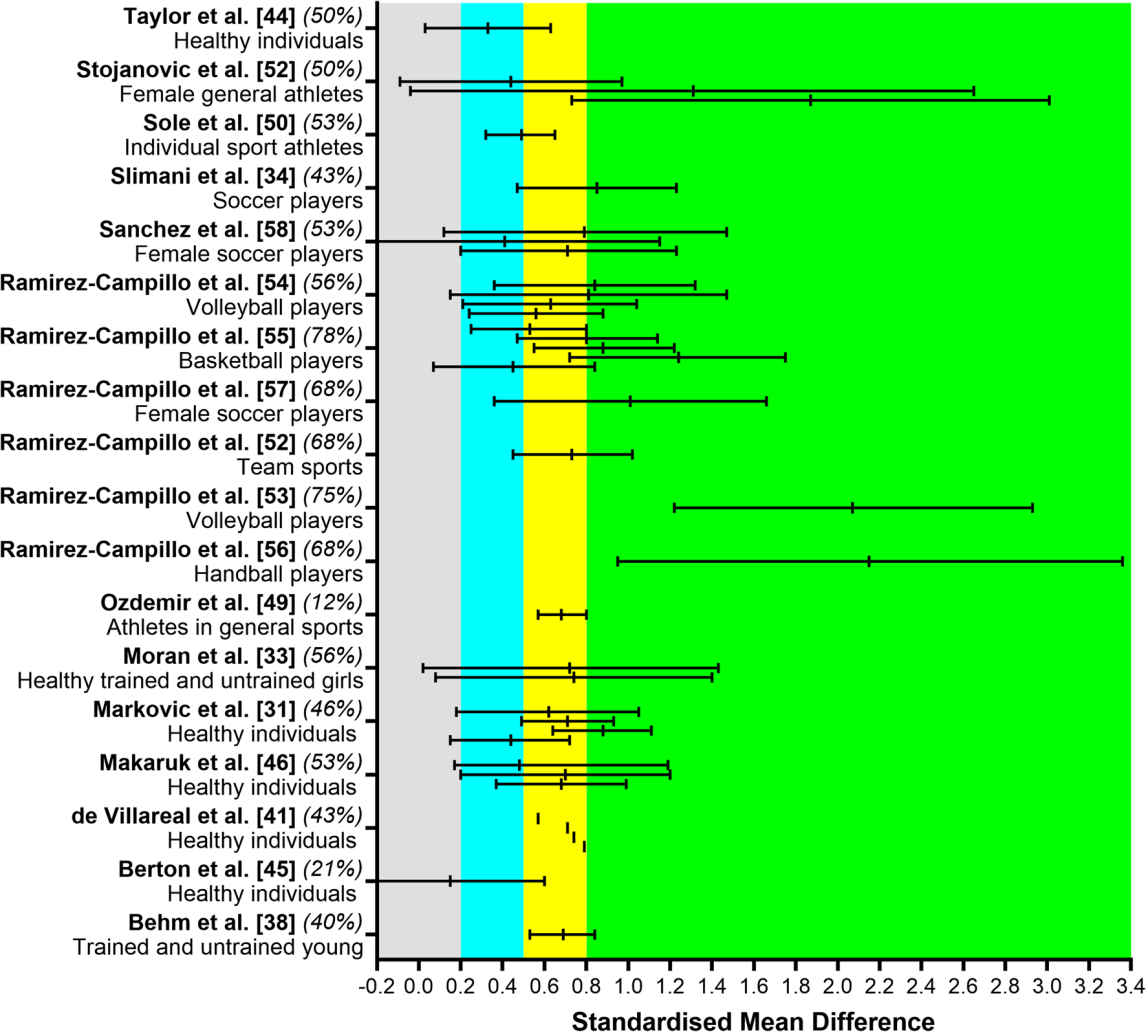
Summary of standardized mean difference and 95% confidence intervals reported in meta-analyses comparing the baseline to post-plyometric training changes on jump performance. Author name and year are followed by the quality of the studies score ranked with the AMSTAR 2. Positive values represent improved performance effects and negative values detrimental effects. Each colored area represents a different magnitude of effect: gray = trivial, blue = small, yellow = moderate, and green = large effects, while the red area represents detrimental effects

Two studies investigated the effects on horizontal jump performance. One study reported a large effect on horizontal jump performance after either horizontal (SMD = 1.05) or vertical plyometric training (SMD = 0.84) [45]. Another study reported unclear effects of plyometric training on horizontal jump distance in basketball players [53]. Detailed SMDs for each study are reported in Table 2, and Fig. 6 summarizes the 18 studies reporting standardized mean difference comparing baseline and post-training values.

### Effect of Plyometric Training on Additional Outcomes

Plyometric training resulted in a small effect on endurance performance for individual sport athletes [48]) and a moderate effect for endurance in female soccer players [56] and for high intermittent running performance in healthy peoples [43]. A large effect was observed on kicking performance in female soccer players [56]. There was also a large effect on dynamic balance, but an unclear effect on static balance in basketball players [53]. Plyometric training improves the Yo-Yo intermittent recovery test when comparing baseline and post-training mean differences [34]. Table 2 presents detailed SMD for each of these studies and variables.

## Discussion

This umbrella review aimed to systematically review the meta-analytical evidence about the effects of plyometric training on physical performance considering different groups, to address the quality, strengths and limitations of the evidence, and to identify current gaps in the literature, which helps in providing suggestions for future research. The most concerning finding from our study is the lack of control group comparisons and the low-to-moderate quality for most of the meta-analyses available in the literature. Therefore, we highlight that the outcomes from these meta-analyses should not be interpreted as level 1 evidence. After summarizing the findings from the available meta-analyses, we observed that plyometric training induces trivial-to-large effects on different physical performance (e.g., jump height, sprint time and muscle strength) for healthy people; enhances performance of athletes from different sports in several motor tasks (e.g., vertical jump height, change of direction, kicking performance and linear sprint time); and induces moderate effects on physical fitness (e.g., power output in lower limbs, change of direction and vertical jump height) in older adults (> 60 years) and young individuals (< 18 years).

### Quality of the Included Meta-analyses

The methodological quality of the included meta-analyses varied from low to high. However, the majority of the studies (~ 75%) were of moderate quality. Based on this, researchers and users should also pay attention to scores within each item for individual studies. Although it is important to pre-register the meta-analysis protocols on a specific platform, only one was registered on PROSPERO (van de Hoef et al. [34]). The reasons are probably related to the older types of review included in this analysis, in which some important criteria were not adopted, e.g. registration on these specific platforms in the health (PROSPERO) and human movement science (TESTEX) areas; in addition, word/table/figure restrictions and/or the absence of databases for supplementary materials would have contributed to this low- to medium-quality bias of umbrella reviews [58, 59].

A very important limitation observed in most of the meta-analyses included in our umbrella review (24 out of 29) was the absence of control groups, and thus, these meta-analyses only included within-group pre- to post-effect sizes. A control group allows the interpretation of the research outcomes removing the influence of possible factors (e.g., direct effect in the specific group). This is crucial when investigating sports performance enhancement because (recreational) athletes follow a training plan during a season, which also influences sports performance. Therefore, the majority of findings presented in this umbrella review should be interpreted with caution. Only five systematic reviews with meta-analysis [30–34] considered the analysis between control versus experimental groups. We strongly recommend that future studies investigating the effects of plyometric training on physical performance adopt randomized controlled trial designs.

### Effect of Plyometric Training on Physical Performance in Non-athlete

Most studies indicate an improvement of vertical jump height, muscle strength and to a lesser extent speed performance in non-athlete people after plyometric training. Considering this population, experimental protocols using plyometric exercises may be a good strategy to optimize health-related aspects [60, 61]. Muscle strength and lower limb muscle power are important capacities for healthy people during daily activities (e.g., walking and climbing stairs), especially when using mechanisms related to the SSC [62].

The vertical jump height was the variable most positively affected by plyometric training according to the included meta-analyses. This variable may be considered as an indicator of muscle power of lower limbs [30, 63, 64], and it is commonly used to verify the effects of plyometric training on physical performance [21, 30, 40–42, 44]. These results are not surprising due to the great specificity, since the same skill (i.e., vertical jump) is used in the testing method and applied in the plyometric training. For the sprint time, a small effect was found for 10-m and 20-m sprint time, a large effect for 30-m sprint time and a small effect for general sprint time. For muscle strength, the effect was unclear, because only one study observed a small effect [46] for healthy individuals and a large effect [41] was observed for non-athletes involved in common sports activities. These results demonstrate a transfer from plyometric training to other physical tasks involving lower limbs [40–42], probably due to neural and muscular adaptations [65]. In addition, it is important to highlight that some experimental aspects might influence the observed effects of the included papers, such as the type of study design, level of experience with plyometric training, and experience in the sport-specific practice.

Upper limb muscle power also demonstrated trivial-to-medium effects of plyometric training. A previous experimental study indicates that plyometric push-ups result in better outcomes compared to non-plyometric push-ups (i.e., dynamic push-ups) [66]. Therefore, neuromuscular adaptations in the upper limbs from plyometric training can be verified, especially in movements involving plyometric push-ups (e.g., medicine ball throw).

### Effect of Plyometric Training on Physical Performance of Athletes in Different Sports

When focusing on different sports, plyometric training induces a large effect on vertical jump height, muscle power and explosive strength (i.e., rate of force development), while a small effect was observed for change of direction. Most meta-analyses including athletes analyzed the effects of plyometric training on physical performance, since maximizing aspects related to sports performance beneficially impacts the training process and competitions [67].

The effects of plyometric training for individual sports demonstrated a medium effect for different variables (e.g., vertical jump height, strength, sprint time and change of direction performances) [49]. When considering team sports, the effects of plyometric training were moderate to large, showing the greater relevance in enhancing performance in this target population. Particularly, for female soccer athletes a high effect was found on vertical jump task [55]. Plyometric training is a practice of physical training with widespread use in the sports context, performed by athletes of different modalities. In this review, larger effect sizes were observed for team sports compared to the other sports groups. Probably athletes from sports such as volleyball, basketball, handball, among others, experience greater adaptation to plyometric training due to the greater specificity of the jumping motor task that is present in training and during the matches.

### Effect of Plyometric Training on Physical Performance in Different Age Groups

This umbrella review indicates that plyometric interventions can enhance physical fitness in children and adolescents beyond a level, which is not exclusively achievable from growth and maturation. In addition, improvements also occurred in middle-aged adults who did not practice sports. Positive effects of plyometric training were found in untrained children and adolescents, especially in vertical jump height, sprint time and muscle strength [37]. Recently, Lesinski et al. [58] observed small-to-medium effects of plyometric training on muscle power of lower limbs in children and adolescent athletes. Other studies also support that plyometric training is an effective training method to improve exercise performance in non-athlete young people [68]. However, moderating factors, such as maturity, sex and age in the youth group, appear to modulate the effects following plyometric training [58, 59]. Thus, future studies should consider these aspects.

In older people, plyometric training improved indicators of muscle power of lower limbs; however, this is supported by only one systematic review with meta-analysis [32]. The aging process is associated with a progressive decline of neuromuscular function, increased risk of falls and injuries related to the impaired functional performance [69–71]. From this perspective, Vetrovsky et al. [72] verified that plyometric training positively affected muscular strength, vertical jump performance, and functional performance (e.g., 30-s sit-to-stand test, figure-of-8 running test, timed up-and-go test, 6-m walk, stair climb) in older adults. Therefore, plyometric training can be considered as a feasible and safe alternative to improve physical fitness in older adults. Future investigations should further explore moderating variables (e.g., age, level of conditioning and body composition).

### Strengths and Methodological Limitations

This umbrella review presented findings on the highest level of the evidence regarding the effects of plyometric training on several physical performance variables in different populations (athletes and non-athletes, male and female) and different age ranges (young and older adult). The majority of the included studies (75%) were of moderate methodological quality when AMSTAR 2 was considered. Finally, this study identified some gaps in the literature to provide guidelines for future research. As a limitation, despite the inclusion of a reasonable number of studies (*n* = 29), few represented females and older individuals. Ultimately, the most important limitation observed in our study was the high prevalence of meta-analysis with the absence of control-group comparisons. This is likely a consequence of low-quality original studies, and this should be addressed in future investigations.

## Conclusion

The current literature presents evidence that plyometric training benefits physical aspects, such as sprint time, change of direction, strength, power and explosive strength. Nonetheless, it is important to bear in mind that most meta-analyses did not include a control condition, limiting the strength of some statements mentioned in papers. This systematic umbrella review unveiled an important weakness of the present research topic. Although several meta-analyses investigated the effects of plyometric training on physical performance outcomes; most of them lack comparisons with control groups and are classified as low-to-moderate quality. It is advised that the outcomes from this umbrella review must not be considered as level 1 evidence. Future research should opt for randomized controlled trials, which will eventually lead to higher-quality meta-analyses. The current evidence, presented by this umbrella review, suggesting that plyometric training may improve a large number of physical fitness-related variables for healthy people and performance for athletes from different sports, and its effects are verified in different age groups and sex, should be taken with caution.

### Supplementary Information

**Additional file 1.** Systematic Search Strategy.

## Data Availability

Not applicable.

## Abbreviations

SSC: Stretch-shortening cycle
PICOS: Population, Intervention, Comparison, Outcome, Study design
AMSTAR 2: A Measurement Tool to Assess Systematic Reviews
SMD: Standardized mean difference
PRISMA: Preferred Reporting Items for Systematic Reviews and Meta-Analyses)
