# Supplementary material for: Effects of Plyometric Training on Physical Performance: An Umbrella Review
Source: Sports Med Open. 2023 Jan 10;9:4. doi: 10.1186/s40798-022-00550-8 (PMC9832201; doi:10.1186/s40798-022-00550-8)
Supplement: Supplementary file 1 — Additional file 1. Systematic Search Strategy. [file 40798_2022_550_MOESM1_ESM.docx]

SYSTEMATIC SEARCH STRATEGY

PUBMED (199 articles)

(("plyometric" OR "jump" OR "countermovement jump"))

Filtered for systematic review or meta-analysis

Web of Science (149 articles)

Search #1 = ALL=("plyometric" OR "jump" OR "countermovement jump")

Search #2 = ALL=(“meta-analysis”)

Search #3 = #2 AND #1

Scopus (64 articles)

( TITLE-ABS-KEY ( "plyometric" OR "jump" OR "countermovement jump" ) ) AND ( TITLE-ABS-KEY ( "meta-analysis" ) ) AND ( LIMIT-TO ( SRCTYPE , "j" ) ) AND ( LIMIT-TO ( DOCTYPE , "ar" ) )

Google scholar (*200 more relevant articles)

(plyometric OR jump OR "countermovement jump") AND ("meta-analysis")
